# Tensiomyography: from muscle assessment to talent identification tool

**DOI:** 10.3389/fphys.2023.1163078

**Published:** 2023-06-26

**Authors:** Dražen Čular, Matej Babić, Damir Zubac, Ana Kezić, Iva Macan, Leonardo Alexandre Peyré-Tartaruga, Francesco Ceccarini, Johnny Padulo

**Affiliations:** ^1^ University of Split, Faculty of Kinesiology, Split, Croatia; ^2^ European Institute for Talents, Education, Research & Development, Split, Croatia; ^3^ Faculty of Kinesiology, University of Zagreb, Zagreb, Croatia; ^4^ Science and Research Center Koper, Institute for Kinesiology Research, Koper, Slovenia; ^5^ Department of Internal Medicine, Center for Integrated Oncology Aachen, Bonn, Cologne, Düsseldorf, University Hospital of Cologne, Cologne, Germany; ^6^ Faculty of Kinesiology, Josip Juraj Strossmayer University of Osijek, Osijek, Croatia; ^7^ LaBiodin Biodynamics Laboratory, Universidade Federal do Rio Grande do Sul, Porto Alegre, Brazil; ^8^ Division of Science, New York University Abu Dhabi, Abu Dhabi, United Arab Emirates; ^9^ Department of Biomedical Sciences for Health, Università degli Studi di Milano, Milan, Italy

**Keywords:** muscle assessment, MHC ratio, talent identification, muscle fiber composition, non-invasive method

## Abstract

**Introduction:** Tensiomyography (TMG) is a non-invasive and cost-effective tool that is gaining popularity in fields such as sports science, physical therapy, and medicine. In this narrative review, we examine the different applications of TMG and its strengths and limitations, including its use as a tool for sport talent identification and development.

**Methods:** In the course of crafting this narrative review, an exhaustive literature search was carried out. Our exploration spanned several renowned scientific databases, such as PubMed, Scopus, Web of Science, and ResearchGate. The materials we sourced for our review included a broad spectrum of both experimental and non-experimental articles, all focusing on TMG. The experimental articles featured varied research designs including randomized controlled trials, quasi-experiments, as well as pre-post studies. As for the non-experimental articles, they encompassed a mix of case-control, cross-sectional, and cohort studies. Importantly, all articles included in our review were written in English and had been published in peer-reviewed journals. The assortment of studies considered provided a holistic view of the existing body of knowledge on TMG, and formed the basis of our comprehensive narrative review.

**Results:** A total of 34 studies were included in the review, organized into three sections: 1) assessing muscle contractile properties of young athletes, 2) using TMG in the talent identification and development process and 3) Future research and perspectives. According to data presented here, the most consistent TMG parameters for determining muscle contractile properties are radial muscle belly displacement, contraction time, and delay time. Biopsy findings from the vastus lateralis (VL) confirmed TMG as a valid tool for estimating the ratio of myosin heavy chain (%MHC-I).

**Conclusion:** TMGs ability to estimate the ratio of %MHC-I has the potential to aid in the selection of athletes with the muscle characteristics best suited for a particular sport, eliminating the need for more invasive procedures. However, more research is warranted to fully understand TMG’s potential and its reliability when used with young athletes. Importantly, the use of TMG technology in this process can positively impact health status, reducing the frequency and severity of injuries and the duration of recovery, and subsequently can reduce drop out rates among youth athletes. Future studies should look at twin youth athletes, as a model capable of discriminating between the influence of hereditary factors *vs*. environmental factors, in therms of muscle contractility and TMG’s potential for instance.

## Introduction

**FIGURE 1:**
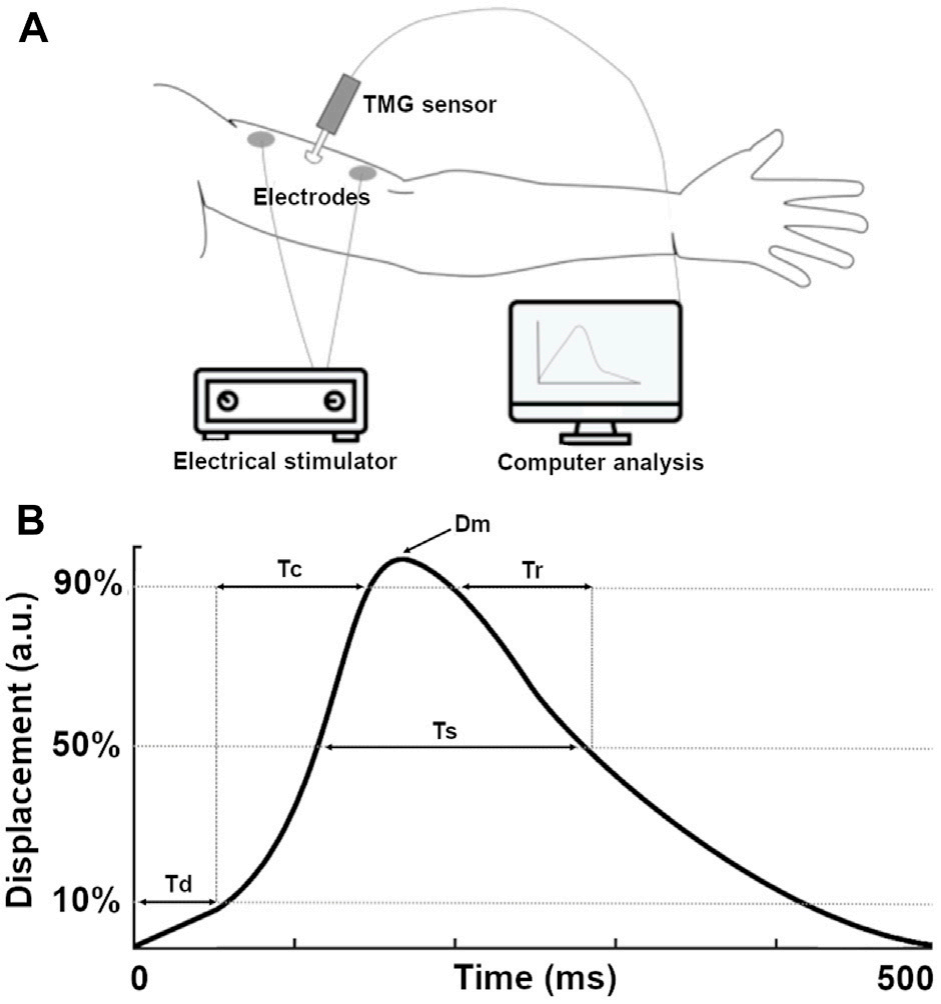
**(A)** Illustration of the tensiomyography (TMG) equipment. **(B)** Example of a classic wave of twitch response with all its parameters.

In the field of muscle contractile properties assessment, tensiomyography (TMG) has emerged as a promising tool over the past two decades. TMG is a non-invasive, mechanomyographic diagnostic tool that employs controlled electrical stimulation to evaluate the contractile properties and muscle tone amplitude of superficial skeletal muscles under isometric conditions (Dahmane et al., 2001; Pišot et al., 2008; Šimunič et al., 2011). Skeletal muscle is stimulated with a single twitch stimulus, through two self-adhesive electrodes symmetrically placed distal and proximal to a high-precision (4 mm) digital transducer, positioned perpendicularly to the muscle surface (Figure 1A). This stimulation results in a displacement-time curve (Figure 1B), from which five parameters are typically extracted: maximal radial displacement (Dm), contraction time (Tc), time delay (Td), sustain time (Ts), and half-relaxation time (Tr).

These parameters provide valuable insights into muscle properties. Dm measures changes in muscle stiffness, muscle mass, and muscle architecture (Pišot et al., 2008; Šimunič et al., 2019). Tc is associated with the proportion of slow-twitch fibers (Dahmane et al., 2001; Dahmane et al., 2005; Šimunič et al., 2011; Zubac and Šimunič, 2017) and muscle contraction velocity (Loturco et al., 2016; Sánchez-Sánchez et al., 2018). Td, the time from the onset of electrical stimulation until 10% of Dm is reached, is interpreted as reaction or activation time (Alentorn-Geli et al., 2015). Ts represents the time between 50% of Dm on both the ascending and descending sides of the curve, while Tr reflects the time at which the muscle displacement reaches 90% of max and falls back to 50% of Dm on the negative slope of the curve.

Human muscle fibers can be classified into different types based on the myosin heavy chain (MyHC) isoform they express. Slow muscle fibers express MyHC type I, while fast muscle fibers express MyHC types IIA and IIX. The most accurate way to identify fiber type is through a muscle biopsy. However, some studies suggest TMG as a useful tool for examining muscle fiber composition. For instance, Šimunič et al. (2011) found a significant correlation between Td, Tc, and Tr, measured by TMG, and the proportion of myosin heavy chain I (%MHC-I) in the VL muscle. This correlation was so strong that their multiple linear model predicted 87% of the %MHC-I variance.

TMG’s ability to examine muscle fiber properties has been applied to a wide range of experimental questions. Šimunič et al. (2018) used TMG in a cross-sectional study desing to assess the contractile properties of the lower limb skeletal muscles in master endurance and power athletes, and age-matched controls. They found that Tc was longer in master endurance athletes compared to nonathletes, a finding also confirmed by %MHC-I data. Moreover, Tc was longest in the BF, followed by GM, and shortest in VL, and these differences were correlated with the proportion of type I fibers. This suggests that the age-related slowing of muscles may be due to a preferential loss of type II fibers. In another study, Zubac et al. (2019) examined the effects of 8 weeks of supervised plyometric training on jumping performance, contractile capacity, and inflammatory response in senior participants. They found no differences in the Tc in VL, and no %MHC-I shift in VL after 8 weeks of plyometric training when estimated from TMG parameters. Still, the information regrading application of TMG among youth athletes are presented by the Slovenian research group (Završnik et al., 2017; Šimunič et al., 2017), thus, further research in this promising area is still warranted.

Using a different approach, Yoshimura et al. (2022) conducted an electromyographic and ultrasound based setup to clarify the link among muscle strength and neural and morphological factors in youth athletes. The results showed a significant correlation between muscle strength and muscle thickness, but not motor unit firing rate. The study concluded that in youth athletes, muscle strength is associated with morphological factors, rather than neural factors, and morphological and neural factors may independently contribute to muscle strength.

Another study (Sözen et al., 2019) used combined electromyographic, mechanimyography and force approach found that electromechanical delay in muscle contraction varies based on sex, age, and physical activity level. Longer delays were observed in women, older individuals, and sedentary participants. These differences were attributed to changes in the mechanical events within the muscle–tendon unit. Overall, compared to other mechanomyography techniques, TMG apparently offers several advantages: is; 1) it minimizes variability and the setup si relatively simple; 2) TMG devices are portable and cost-effective, which make them suitable for a wide range of clinical and research settings.

The purpose of this paper is to critically evaluate the application of TMG in sports science and medicine, its strengths and limitations, with particular emphasis on TMG as a useful tool for identifying sports talent.

## Methods

### Literature search

In the course of crafting this narrative review, an exhaustive literature search was carried out. Our exploration spanned several renowned scientific databases, such as PubMed, Scopus, Web of Science, and ResearchGate. The materials we sourced for our review included a broad spectrum both experimental and non-experimental articles (*n*. 34), all focusing on TMG. Researchers selected specific criteria for scientific paper selection: papers published in English, open-access paper/full-text papers, including information about TMG, include a variety of sports, including differentiation of TMG parameters**,** include details on the utility of TMG, include information about the validity of TMG. The search strategy was defined following the aim of the investigation. The authors have selected relevant terms for this research, which they used as search terms in selected databases. Used terms are presented next: Tensiomyography AND talent identification OR TMG AND talent identification, Tensiomyography AND talent initiation OR TMG AND talent initiation, Tensiomyography AND talent selection OR TMG AND talent selection, Tensiomyography AND talent detection OR TMG AND talent detection, Tensiomyography OR TMG, Tensiomyography AND %MHC-I OR TMG AND %MHC-I, Tensiomyography AND fiber OR TMG AND fiber, Tensiomyography AND type II OR TMG AND type II, Tensiomyography AND fast-twitch OR TMG AND fast-twitch, Tensiomyography AND fast fiber OR TMG AND fast fiber.

The experimental articles featured varied research designs including randomized controlled trials, quasi-experiments, as well as pre-post studies. As for the non-experimental articles, they encompassed a mix of case-control, cross-sectional, and cohort studies. Importantly, all articles included in our review had been published in peer-reviewed journals. The assortment of studies considered provided a holistic view of the existing body of knowledge on TMG, and formed the basis of our comprehensive narrative review.

## Results and Discussion

This Chapter is organized into three sections: 1) TMG application in assessing muscle contractile properties among young athletes, 2) Application of TMG in the process of talent identification and development 3) Future research and perspectives.

### TMG application in assessing muscle contractile properties among young athletes

In many sports, intensive training at a young age is considered to be a necessary step for achieving higher levels of performance. For example, elite female gymnasts begin training at a young age, when they are 5 or 6 years old. They undergo progressive training in terms of intensity and volume, often training for 20–30 h per week year-round throughout their childhood and adolescence. This intense training allows them to reach their peak performance around the age of 16, and rarely their athletic careers continue beyond their 20 s. Therefore, it is important to monitor training programs in young populations, in order to prevent excessive high-intensity activities and reduce the risk of injury, illness, and non-functional overreaching in young athletes.

Is TMG a reliable tool to examine the muscle proprieties of young athletes? So far few studies have investigated this aspect. One possible reason for this lack of research may be the additional ethical considerations that must be taken into account when working with underage athletes. However, the few existing studies seem to confirm that TMG can be used with young non-athlete populations. For instance, Pregelj and Šimunič (2018) investigated the effects of electrical muscle stimulation (EMS) on contractile properties of children aged 9 to 14. TMG resulted sensitive enough to detect a decreased in Dm caused by the EMS-induced treatment. Završnik et al. (2017) examined the relationship between running speeds and Tc in children aged 8 to 13, in a 5-year study. They found a strong negative correlation between Tc of biceps femoral and running velocity. Other studies found found that regular participation in sports was associated with shorter BF Tc in both boys and girls (age higher than 12y), while there was no effect of sports participation on the VL Tc (Šimunič et al., 2017).

It is important to note that other studies have raised doubts about the effectiveness of TMG in assessing muscle properties in young athletes. For example, a pilot study by Wiewelhove et al. (2017) found that TMG parameters were not sensitive enough to detect significant changes in muscular performance in elite youth athletes. However, it is important to note that pilot studies are generally small in scale, and they are not representative of the population as a whole, so it is possible that the results of Wiewelhove et al. (2017) may not be generalizable to other populations.

It is important to note that TMG is a relatively new noninvasive technology therefore, without large ethical considerations; anyway more research needs to be conducted in order to fully understand its effectiveness in assessing muscle properties in young athletes.

### TMG and talent identification

Talent is a frequently used term describing a natural aptitude or ability that someone has for a particular activity or task (Till and Baker, 2020). Different definitions of talent have been proposed. For instance, Brown (2002) described talent as a special and natural ability for achievement or success. In sports science, talent identification involves identifying young athletes with the potential to become elite athletes (Brown, 2002). Nowadays, finding, recruiting, and retaining talented athletes is a challenge, faced by sports clubs and national federations globally (Koz et al., 2012). For this reason, many efforts have been done to explore various methods to identify and develop talent in sports, and different approaches have been proposed. Current models are based on skills and expertise (e.g., McDermott, et al., 2015), statistical models (e.g., Höner, et al., 2015), current performance (e.g., Allen, et al., 2014) or even genetic testing or physiological assessments (e.g., Breitbach, et al., 2014). New co-twin study (Babić et al., 2023) as one of the few twin studies conducted on champion-level athletes show that muscle contractile properties of VL have the greatest hereditary potential, followed by VM and RF. Nevertheless, the results of the examined muscle contractile properties suggest that scores of Td, Tc and Vc, which represent measures of relative and absolute speed/velocity capabilities in lower-limb muscles, have great hereditary potential. Onwards, this research revealed that Td, seems to be the most inheritable among measured properties, followed by muscle tissue capability to produce contraction in a fraction of time, and thirdly the Vc as equivalent to the applied muscle contraction measures (Counter movement—squat jumps). However, identifying and developing talent in sports remains a global challenge, and current models for talent detection are often inadequate. Furthermore, a recent study (Padulo et al., 2023) brings new insights into talent detection and development topics and highlights the significance of evaluating the physiological factors impacting performance in athletes who have undergone specific training and how different muscle fiber types contribute to the energy cost and running economy in these athletes and how it affects their performance. This information, in the contects of TMG potential, also could be useful in talent development process.

May TMG represent an effective tool for talent identification? In this regard, TMG may help to identifies individuals with superior athletic capacity determined by their muscle characteristics. Indeed, the variation in muscle fiber composition could predict differences in physical performance among individuals (MacDougall et al., 1998; Fry, 2004). Type I muscle fibers specialize in endurance activities, as long-distance running, whereas type IIx muscle fibers specialize in fast and powerful movements, such as sprinting and weightlifting (MacDougall et al., 1998; Fry, 2004). In light of this, the estimation of MHC—I *vs*. MHC—II ratio (%) from TMG, may be useful for selecting people whose muscle characteristics are particularly indicated for a specific sport activity.

The application of TMG methodology in the process of talent detection can be highly beneficial, especially when considering the results of the study Gillen et al. (2022). The study examined peak torque (PT), mean power (MP), EMG amplitude, MMG amplitude, and neuromuscular efficiency across the velocity spectrum in children *versus* adolescents. The findings of the study suggest that there are significant differences in PT, MP, and neuromuscular efficiency between children and adolescents. This information could be valuable in the talent detection process, as it provides insight into the development of neuromuscular function during dynamic movements. TMG could be used to monitor these parameters over time, providing a non-invasive and objective measure of muscle function and development. This could help identify young athletes who show early signs of superior transmission efficiency (Peyré-Tartaruga and Coertjens, 2018) and, consequently, neuromuscular effectiveness potentially indicating a natural talent for certain sports or activities.

Although the use of TMG for talent identification appears promising, further research is needed to fully understand its potential. Two main issues need to be addressed to fully evaluate its usefulness. Firstly, TMG should have the ability to identify “talent” at the beginning of the sports career. However, TMG’s capability of describing muscle fiber composition has been inferred from adult samples, and this may raise questions about its reliability when used with young athletes.

Secondly, previous literature lacks information about the optimal time frame for conducting a talent assessment by using TMG. In other words, to utilize TMG as a talent identification tool, it is crucial to establish what is the best time, during puberty or adolescence, to conduct a muscle examination in able to predict the future performance of athletes. The human body experiences significant physical and hormonal changes as it transitions from childhood to adulthood, and muscle contractile properties can be influenced by the presence and concentration of hormones, such as testosterone and estrogen, which play a role in muscle development (Kraemer et al., 1990; Bryner et al., 1999). Moreover, muscle contractile properties seem to depend on pubertal maturing, which occurs at different ages according to sex. Girls enter puberty earlier than boys, probably because of the hormone Leptin, which is proven to trigger puberty onset. Leptin, produced in adipose tissue, directly correlates with body fat and body mass index (Garcia-Mayor et al., 1997), and it is responsible for the start of the puberty process, where there is a parallel growth of leptin and estradiol, follicle stimulating, and luteinizing hormones among girls. At the same time, the rise of testosterone is inversely correlated with leptin levels among boys. These aspects should be taken into consideration by future studies investigating the use of TMG in talent identification.

In the talent detection process, future research could focus on a comparative study, such as, per example: “The Use of Tensiomyography in Talent Detection - A Twin Study”. This study would aim to investigate the muscle contractile properties of identical twins who engage in different levels of physical activity/sport. The proposed study would involve pairs of identical twins, with one twin from each pair regularly participating in sports training and the other as non-athletic lifestyle. TMG would be utilized to measure various parameters of muscle contraction, and these measurements would be compared between the two groups. The goal of this future research would be to ascertain whether regular sports training results in significant differences in TMG parameters.

As a concluding remark, it is important to note that even if TMG could help to identify the optimal muscular predisposition in specific sports activities, talent identification is a complex process that involves multiple factors beyond just muscle contractile properties and fiber composition. Previous literature has pointed out the importance of considering talent as a holistic construct including various factors such as physical, psychological, and social aspects (e.g., Vaeyens et al., 2006).

## Future research and perspectives

A main strength of this study is that it sheds new light on the use of TMG in the identification and development of sport talents. TMG is, as we mention before, indubitably a promising noninvasive research tool with potential of distinguishing inherently gifted athletes for specific sports from other athletes. In addition, TMG eliminates the need for invasive biopsy procedures and avoids the ethical issues associated with these procedures. It has potential to be used for estimation and monitor muscle fiber composition in young athletes during growth and development. Overall, this is the one of the first studies addressing the relationship between talent identification and non-invasive TMG estimation of muscle fiber composition in adolescent athletes. Despite ethical issues related to the process of sports talent detection, it is important to emphasize that the use of TMG technology in this process can certainly positively impact health status, namely reducing the frequency and severity of injuries and the duration of recovery. Although it does not explicitly mention TMG methodology, Mostafavi et al. (2021) highlight the use of technology that can “*alert*” athletes to the risk of injury.” Limitations of the study include the heterogeneity of the analised studyes and the selection of participants (including young athletes, professional athletes, sedentary, aged, and diseased individuals of both sexes), which make all meta-analysis complicated and rather impossible. In addition, only a limited number of studies examined the applicability of TMG measurements in adolescent athletes, which limits the ability to draw meaningful conclusions from the analyzed literature. Further investigations should include a greater sample size of elite youth athletes, non-athletes and other significant groups (twins) as well as the establishment of new methods to assess precise heritability scores and fiber composition scores noninvasively. Nonetheless, preliminary results suggest that TMG is a promising noninvasive tool that can be used to estimate muscle fiber composition and thus genetic predisposition in talent acquisition, identification, selection, and development.

## Conclusion

TMG is a valuable tool for muscle contractile properties evaluation, providing accurate results in medical and sports contexts. The non-invasive nature of the method offers several advantages compared to conventional invasive techniques: 1) it is a non-invasive and efficient method, providing accurate information about muscle properties without disrupting the daily routine of athletes; 2) it minimizes variability and the setup is relatively simple; 3) TMG devices are portable and cost-effective, which make them suitable for a wide range of clinical and research settings. However, more research is necessary to fully understand TMG’s potential and limitations, especially in young individuals and for talent identification. Further investigation is needed to determine the reliability of TMG when used with young athletes and to consider ethical implications to avoid discrimination in the talent detection process. Future areas of research could include the optimal time frame for TMG assessments and the impact of hormones and adolescent development on muscle fiber composition. In addition, studies in specific populations, such as monozygotic and dizygotic twins, may provide new insights into the extent to which TMG outcomes are influenced by genetic and/or environmental factors.
